# Bilateral Maxillary Sinus Hypoplasia

**DOI:** 10.1155/2014/148940

**Published:** 2014-12-08

**Authors:** Sachin Khanduri, Sumit Agrawal, Saakshi Chhabra, Swati Goyal

**Affiliations:** ^1^Era's Lucknow Medical College, C-149, Indiranagar, Lucknow 226016, India; ^2^Era's Lucknow Medical College, Room No. B-14, P.G. Boys' Hostel, Hardoi Road, Sarfarazganj, Lucknow 226003, India; ^3^Era's Lucknow Medical College, P.G. Girls' Hostel, Hardoi Road, Sarfarazganj, Lucknow 226003, India

## Abstract

Maxillary sinus hypoplasia (MSH) is an uncommon abnormality of paranasal sinuses noted in clinical practice. Computed tomography (CT) scan helps in diagnosing the anomaly along with any anatomical variation that may be associated with it. MSH is usually associated with other anomalies like uncinate process hypoplasia. Three types of MSH have been described. Type 1 MSH shows mild maxillary sinus hypoplasia, type 2 shows significant sinus hypoplasia with narrowed infundibular passage and hypoplastic or absent uncinate process, and type 3 is cleft like maxillary sinus hypoplasia with absent uncinate process. CT and endoscopic examination usually complement each other in diagnosing MSH.

## 1. Introduction

MSH is an uncommon abnormality that comes across in clinical practice. It has been reported in 1.73% to 10.4% of patients with sinus symptoms [1]. However it sometimes is asymptomatic and is diagnosed on radiological evaluation. Maxillary sinus develops in 3rd month of fetal life as mucosal evagination of middle meatus of nasal cavity with simultaneous resorption of maxillary bone. Volume of maxillary sinus at birth is 6–8 mm^3^. It increases by 2 mm^3^ per year in vertical and lateral dimension and 3 mm^3^ in anteroposterior dimension. At 10 years lower boundary of sinus is at the level of nasal cavity floor [2]. As permanent dentition occurs extension of sinus occurs 4-5 mm below the level of nasal cavity inferiorly [3]. Etiology of MSH includes both embryological and acquired causes like trauma or infection causing arrest of sinus pneumatisation [4] (Table 1).

## 2. Case Report

**Figure 1:**
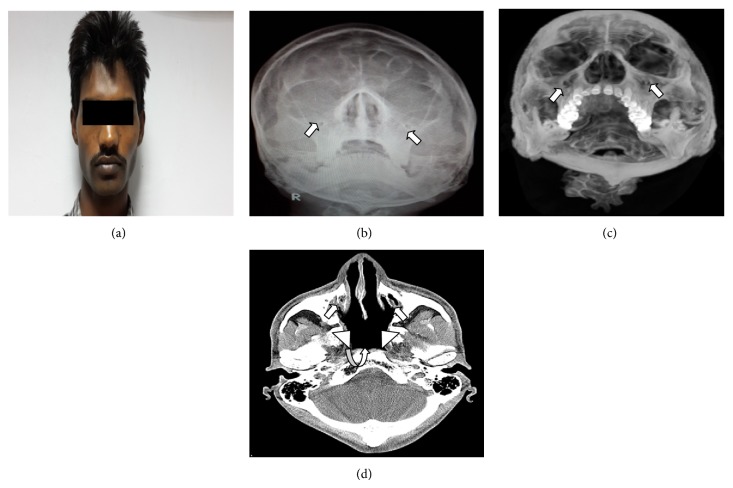
25-year-old male with recurrent headache. Face profile of patient (a). Opacification of bilateral maxillary sinuses (arrow) on plain radiograph (b) with loss of pneumatisation (arrow) on 3D CT reconstruction paranasal sinuses (c). Cleft like bilateral maxillary sinuses (arrow) and absent uncinate process (arrowhead) and enlargement of nasal fossa (curved arrow) on axial section plain CT paranasal sinuses (d).

**Figure 2:**
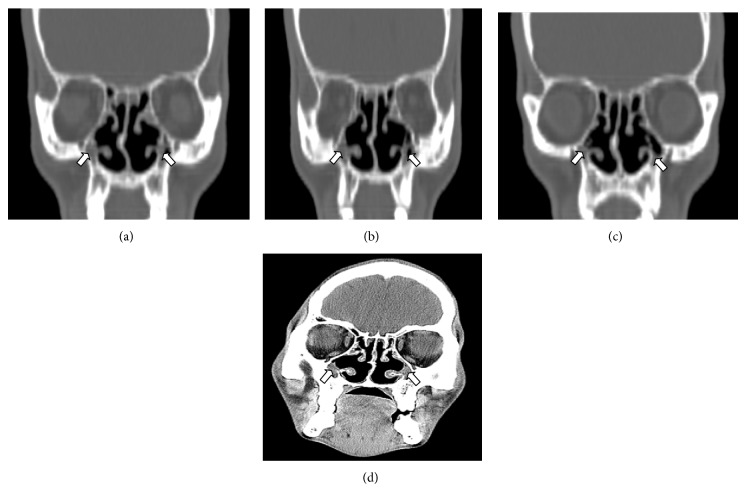
25-year-old male with recurrent headache. Coronal sections plain CT paranasal sinuses showing cleft like bilateral maxillary sinuses with loss of pneumatisation (arrow).

25-year-old male presented to otorhinolaryngology out-patient department with chief complaints of nasal discharge and headache on and off since childhood. Neurological examination was carried out and was reported to be normal. Patient was referred to radiodiagnosis department where the patient was taken up for X-ray paranasal sinuses water's view which showed opacification of bilateral maxillary antrum (Figures 1 and 2). As the X-ray findings were inconclusive provisional diagnosis of sinusitis was made and antibiotic treatment was started. Patient was unresponsive to antibiotic treatment. After these CT paranasal sinuses were done on SIEMENS machine mode SOMATOM. The study revealed bilateral opacified and hypoplastic cleft like maxillary sinuses with narrowing of infundibular passage with absent uncinate process with enlarged nasal fossae; however the orbits appeared normal. On the basis of CT findings and patient presentation diagnosis of bilateral hypoplastic maxillary sinuses type 3 [5] was made.

## 3. Discussion

Causes of maxillary sinus hypoplasia are trauma, infection, surgical procedure, irradiation, and congenital anomaly. Congenital anomaly such as Treacher Collins syndrome is associated with unilateral maxillary sinus hypoplasia.

### 3.1. Clinical and Imaging Findings

Classification of MSH has been described in previous studies. Bolger et al. classified MSH into three types. Type 1 MSH shows mild maxillary sinus hypoplasia, type 2 shows significant sinus hypoplasia with narrowed infundibular passage and hypoplastic or absent uncinate process, and type 3 is cleft like maxillary sinus hypoplasia with absent uncinate process [5].

Sirikci et al. classification included orbital involvement into the above classification [6]. Ipsilateral orbital enlargement was included in type 2 and type 3 Bolger et al. classification.

## 4. Teaching Point

CT paranasal sinuses should be included in diagnosis protocol because it helps us in reaching the diagnosis earlier and with accuracy as we saw in this case where plain radiograph was inconclusive and patient was misdiagnosed as a case of sinusitis; however after CT the correct diagnosis of maxillary hypoplasia was made. Also, CT helps us in diagnosing any associated anomaly that may be present which turns out to be an important guide in deciding the course of surgery and to avoid any unforeseen surgical complications.

## Figures and Tables

**Table 1:** Summary table.

Etiology	Embryological and acquired causes like trauma or infection causing arrest of sinus pneumatisation

Incidence	1.73% to 10.4% of patients with sinus symptoms

Gender ratio	No sex predilection

Age predilection	No age predilection

Risk factors	None

Findings on imaging	X-ray opacification of sinuses, CT-cleft like sinus with absent uncinate process
